# Targeting CK2 in cancer: a valuable strategy or a waste of time?

**DOI:** 10.1038/s41420-021-00717-4

**Published:** 2021-10-29

**Authors:** Mauro Salvi, Christian Borgo, Lorenzo A. Pinna, Maria Ruzzene

**Affiliations:** 1 Deptartment Biomedical Sciences, University of Padova, Padova, Italy; 2 CNR Neuroscience Institute, 35131 Padova, Italy

**Keywords:** Kinases, Cancer, Cell signalling, Phosphorylation

## Abstract

CK2 is a protein kinase involved in several human diseases (ranging from neurological and cardiovascular diseases to autoimmune disorders, diabetes, and infections, including COVID-19), but its best-known implications are in cancer, where it is considered a pharmacological target. Several CK2 inhibitors are available and clinical trials are underway in different cancer types. Recently, the suitability of CK2 as a broad anticancer target has been questioned by the finding that a newly developed compound, named SGC-CK2-1, which is more selective than any other known CK2 inhibitor, is poorly effective in reducing cell growth in different cancer lines, prompting the conclusion that the anticancer efficacy of CX-4945, the commonly used clinical-grade CK2 inhibitor, is to be attributed to its off-target effects. Here we perform a detailed scrutiny of published studies on CK2 targeting and a more in-depth analysis of the available data on SGC-CK2-1 vs. CX-4945 efficacy, providing a different perspective about the actual reliance of cancer cells on CK2. Collectively taken, our arguments would indicate that the pretended dispensability of CK2 in cancer is far from having been proved and warn against premature conclusions, which could discourage ongoing investigations on a potentially valuable drug target.

## Introduction

CK2 is a protein kinase implicated in many human diseases [1]. Recently, it has been found relevant also in the viral infection by SARS-CoV-2 (severe acute respiratory syndrome coronavirus 2) and its exploitation as a COVID-19 target is under investigation [2, 3]. The best-known functions of CK2, however, are in cancer, where it is frequently overexpressed and currently considered a valuable pharmacological target [4–7]. Several CK2 inhibitors are available and two of them are in clinical trials [8, 9].

A recent publication [10] reported that a newly developed CK2 inhibitor, SGC-CK2-1, is much more selective than previously employed CK2 inhibitors, but surprisingly is poorly effective in reducing cell growth in different cancer lines. This prompted some investigators to question the suitability of CK2 as a broad anticancer target [11], attributing the efficacy of CX-4945, the commonly used clinical-grade CK2 inhibitor [9], to its off-target effects.

We think that such a conclusion is premature and eventually deceptive, as it unduly nullifies valuable efforts made so far by many researchers, discouraging investigations in a field that instead still appears quite promising. Our point of view is mainly based on three pieces of evidence, as described below: (1) a survey of data in the literature about the effects produced in tumor cells by targeting CK2 with non-pharmacological tools; (2) the abnormally high CK2 levels in many kinds of tumors; and (3) an in-depth analysis of the available data on SGC-CK2-1, which leads to an interpretation different from the one provided in ref. [11].

## Non-pharmacological CK2 targeting produces antiproliferative and pro-apoptotic effects in cancer cells

**Fig. 1:**
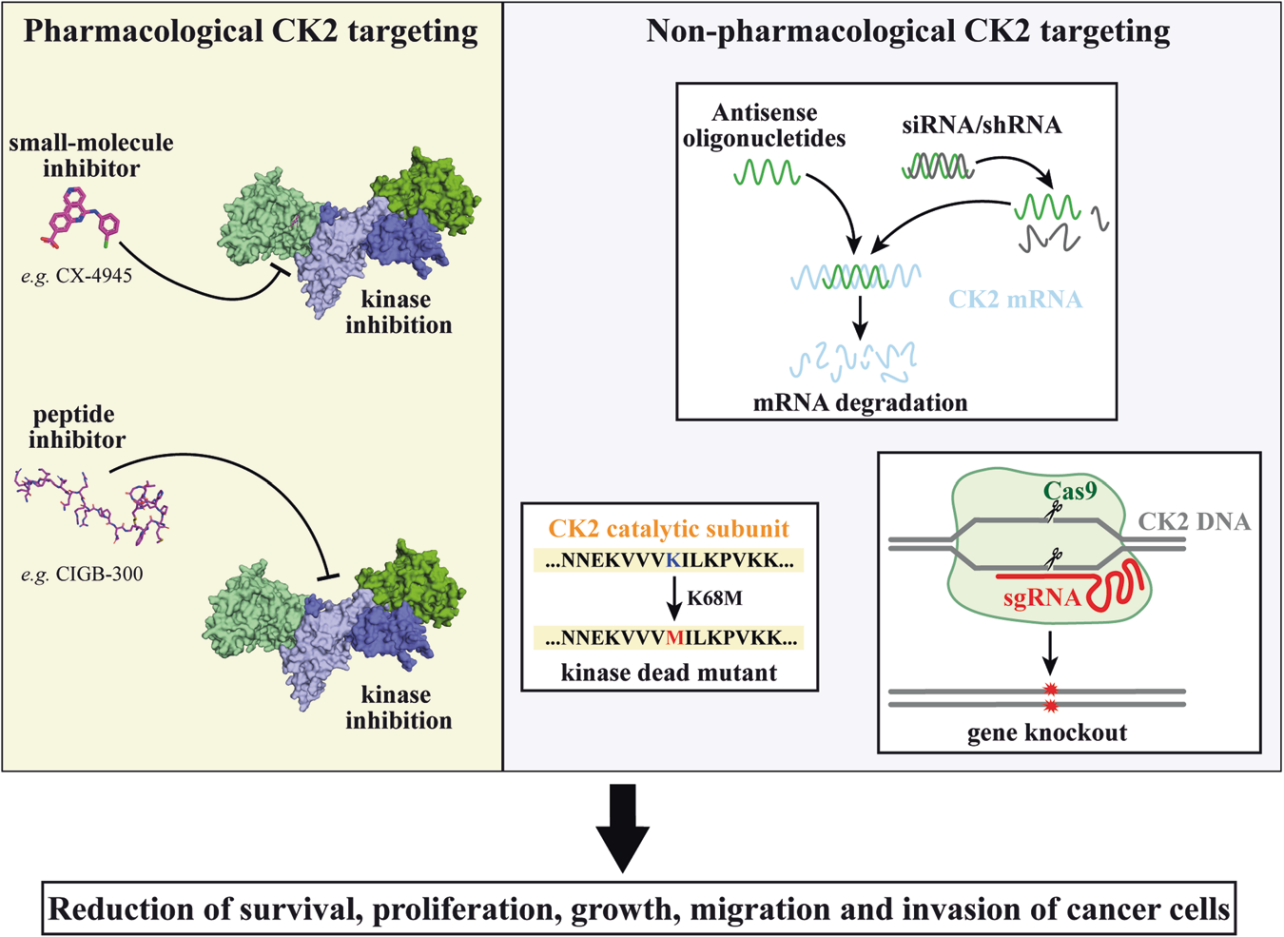
Pharmacological and non-pharmacological approaches that have been used to target CK2 in cancer cells. The left side shows the major pharmacological targeting strategies, represented by ATP-competitive small molecule inhibitors (as the clinical-grade inhibitor CX-4945, upper) or peptide-based inhibitors (as the clinical-grade compound CIGB-300, bottom). The right side schematically shows the most common non-pharmacological approaches to target CK2: the mRNA downregulation by antisense oligonucleotides or shRNA/siRNA (upper), the expression of kinase-dead mutants (bottom, left), and the gene knockout by CRISPR/Cas9 technology (bottom, right). All strategies reduce the survival and attenuate the oncological features of cancer cells.

The functional relevance of CK2 in regulating cancer cell proliferation and survival has been suggested not only by observing the effect of different structurally unrelated CK2 inhibitors whose specificity may be questionable, but also by using a variety of non-pharmacological treatments to control kinase expression and, consequently, kinase activity (Fig. 1). Non-pharmacological approaches, ranging from antisense oligonucleotides or small interfering RNA (siRNA) to overexpression of kinase-death mutants, have been applied since the beginning of the investigation on the role of CK2 in cancer. Several independent reports proved that, when the kinase amount was transiently decreased by regulating the expression level of its subunits, cancer cell proliferation and survival were reduced, as well as their migration and invasiveness. Although the effects of downregulating an individual CK2 subunit on cell proliferation/viability are variable, according to the cell type, they have been observed in a wide variety of cancer cells. In glioblastoma cells, siRNAs treatment against both *α*- and *β*-subunit of CK2 decreases cell viability [12, 13]; in colorectal cancer cells, the CK2α targeting by siRNA inhibits cell proliferation, promoted cell senescence, and inhibit cell migration and invasion [14]; in breast cancer cells, knockdown of CK2α inhibited cell proliferation, migration, and invasion [15]; in hepatoma cells, knockdown of CK2α inhibits cell proliferation, cell migration, and invasion, and induces apoptosis [16]; in non-small cell lung cancers, CK2α targeting induces apoptosis [17]; siRNA against CK2α in a chronic lymphocytic leukemia cell line resulted in a significant decrease in cell viability [18]; in multiple myeloma cell lines, CK2α targeting induces apoptosis [19]; and in human osteosarcoma cells, knockdown of CK2α or CK2β inhibited the proliferation [20].

Concomitantly, the downregulation of CK2 subunits increases the sensitivity to different chemotherapeutic agents [21–32].

To be mentioned, finally, the effects of CK2 downregulation in xenograft cancer models. The in vivo delivery to cancer cells of specific CK2-siRNA targeting both the catalytic subunits by nanocapsules has been found effective in inhibiting tumor growth and inducing cell death in xenograft models of head and neck squamous cell carcinoma [33, 34], prostate cancer [35, 36], and breast cancer [37]. Moreover, in a U87-MG-derived glioblastoma xenograft model, expressing an inducible short hairpin RNA targeting CK2α, a significant reduction in tumor size and growth was observed in parallel with the reduction of CK2α expression [38].

With the advent of the CRISPR/Cas9 tool, it has been possible to produce viable knockout cancer cells for each CK2 catalytic subunit. Even if the production of these cells proved that the single individual catalytic CK2 subunit is not strictly required for cancer cell survival, these cells generally show a reduction in cell proliferation, motility/invasiveness, and an increase in sensitivity against chemotherapeutic agents when compared with wild-type cells [39–42]. Again, these effects are variably pronounced, according to the cell type and the target subunit gene. Any attempt to produce tumor cells fully devoid of both CK2 catalytic subunits has failed so far, suggesting that a complete absence of CK2 activity is incompatible with cell life.

These data by themselves unambiguously demonstrate the reliance of cancer cells on CK2 for their survival by an approach fully independent of pharmacological treatment, thus circumventing the hypothesis that the effects of CX-4945 are mediated by its nonspecific targets.

## Abnormally elevated CK2 in cancer cells: a neglected clue

An aspect of malignancy that has not been duly considered in refs. [10, 11] is the abnormally high CK2 expression/activity found in a wide variety of cancer cells [4, 43–46], consistent with the view that such a genetic trait is selected by transformed cells, because it maintains/potentiates the tumor phenotype. Although such an argument is per se coincidental in nature, a cause–effect relatedness between elevated CK2 and malignancy is disclosed by observing the pro-apoptotic efficacy of CK2 inhibitors, which is more accentuated in tumor cells as compared with non-tumor ones (see ref. [47]). Such a scenario is hardly explainable by purported off-target effects of CK2 inhibitors unless we assume the implausible theory that these effects are invariably more pronounced in cancer cells.

## There is not sufficient evidence of a lower antitumor efficacy of SGC-CK2-1 compared to CX-4945

The claim that CK2 is dispensable in cancer cells [11] is mostly based on the comparison between the effects induced by a very specific inhibitor (SGC-CK2-1) [10] and those of the less selective and commonly used inhibitor, CX-4945 [9]: as “SGC-CK2-1 does not demonstrate significant antiproliferative activity against a panel of 140 different cancer cell lines,” [10] the authors conclude that the antitumor properties of CX-4945 (as reported by hundreds of studies, reviewed in ref. [48]) are mediated by unspecific targets.

However, it is noteworthy that when Wells et al. [10] compared SGC-CK2-1 and CX-4945 for their antiproliferative effects, they found very similar half maximal inhibitory concentration (IC_50_) values. The direct comparison has been performed only on HCT116 cells, showing IC_50_ > 10 μM for both compounds, calculated from a ProQuinase proliferation assay. Surprisingly, by a different method, the same work reports 1.9 and 2.2 μM IC_50_ values for SGC-CK2-1 and CX-4945, respectively. Therefore, there is a discrepancy between the efficacy of SGC-CK2-1 in the same cell line depending on the method, but the same discrepancy is mirrored by CX-4945. From these data, the assignment of the CX-4945 effects to its off-targets appears inconsistent.

Of course, the efficacy of an inhibitor in cells primarily depends on its inhibitory potency in vitro. The in vitro inhibition of CK2 (in terms of Ki or IC_50_) has never been reported for SGC-CK2-1, but a nanoBRET assays (for the compound binding to CK2) indicates IC_50_ in the nM range (36 nM for CK2α and 16 nM for CK2α’) [10]. These values are in line with those of CX-4945 (Ki = 0.38 nM, IC_50_ 1 nM) [9], which is, in case, even more potent.

The inhibitory efficacies of SGC-CK2-1 and CX-4945 are similar also towards endogenous CK2 in cells. The concentrations of SGC-CK2-1, which are effective in cells, are in the sub-micromolar range [10]. Also for CX-4945, we found a very strong inhibition in cells at sub-micromolar concentrations [49], at variance with what was reported by Wells et al. [10], who found CX-4945 effects at higher concentrations, probably due to the artificial system employed, with CK2 ectopically overexpressed in cells. Direct experimental comparison on endogenous CK2 in HCT116 cells is not possible, as in ref. [10] a single, very high concentration of CX-4945 was used (10 μM, completely inhibitory).

It can be argued that, given the inhibitory values, a cellular response could have been expected at lower concentrations and this has probably driven the conclusions on the poor antiproliferative effects of SGC-CK2-1 [10, 11]. However, as discussed in ref. [49], this discrepancy in the concentration range required for CK2 inhibition vs. antiproliferative efficacy is also observed for CX-4945 (Ki towards recombinant CK2 of 0.38 nM [9] and antiproliferative effect in the μM range [49]).

In summary, our scrutiny suggests that the cellular efficacy and the antiproliferative effects of SGC-CK2-1 roughly correspond with what is expected in comparison with CX-4945, thus challenging the conclusion that the effects induced by CX-4945 must be ascribed to its unspecific targets.

It should be also noted that very recently, a chemical modification of CX-4945 has given rise to a CK2 inhibitor more specific than CX-4945, but with improved, rather than impaired, antitumor properties [50].

It is also worth noting that, in some cases, the specificity of the cellular effects induced by a chemical inhibitor has been confirmed by transfecting a CK2 mutant insensitive to the inhibitor (because bearing mutations of sites crucial for the compound binding) and showing that this prevented the cell responses to the inhibitor (e.g., see ref. [19]).

We agree that SGC-CK2-1 is a very powerful tool, probably representing the most selective CK2 inhibitor available, which means the first-choice compound for dissecting the CK2 cellular functions. However, further investigation is necessary to disclose its potential effects in cancer. CK2 controls practically all cancer hallmarks [51], whereas the efficacy of SGC-CK2-1 has been evaluated only on proliferation, so far. Especially important, the induction of apoptosis in response to SGC-CK2-1 has not been thoroughly evaluated. Moreover, individual substrates of CK2 have been associated with specific features of cancer cells. Wells et al. [10] evaluated the endogenous CK2 residual activity by Akt phospho-Ser129. Although this site is widely considered a reliable reporter of cellular CK2 activity [52], there are individual substrates that mediate CK2-specific functions and whose phosphorylation state responds to a variable extent to the same inhibitor concentration, due to different reasons. We have already observed and discussed this phenomenon [49, 53]. Therefore, the phosphorylation level of CK2 physiological targets other than Akt Ser129 should be evaluated in response to SGC-CK2-1.

In summary, on one hand, additional tumor features, besides cell proliferation, should be analyzed before drawing conclusions about the antitumor properties of a given CK2 inhibitor. On the other hand, the inhibitor concentrations used for cell treatment are not sufficient to completely abolish CK2 activity and to prevent the phosphorylation of a subset of critical CK2 substrates. Pertinent to this may be the observation that C2C12 myoblasts fully deprived of both CK2 catalytic subunits survive, thanks to the presence of minimal traces of CK2 activity, attributable to a deleted form of the α’-subunit still able to generate a number of phospho-sites unambiguously attributable to CK2 [53]. It is noteworthy that these phospho-sites differentiate from those of wild-type cells for their different susceptibility to two CK2 inhibitors (manuscript in preparation).

Concerning the variable sensitivity of different cell lines to CK2 inhibition, as reported by Wells et al. [10] in the case of SGC-CK2-1, it should be mentioned that the same phenomenon has been observed in several studies also with other CK2 inhibitors (e.g., see ref. [54]) and we can speculate about peculiar features that cancer cells must acquire in order to become more resistant to the reduction of CK2 activity/amount. In general, blood cancer cells seem to display a higher sensitivity to CK2 inhibitors (with the possible speculation, however, about the higher capacity for the compounds to reach intracellular CK2 in cells growing in suspension, compared to adherent cells). It could be speculated that there might be a genetic background, which is shared by all very sensitive (or very resistant) cells. A p53-dependent induction of apoptosis by CK2 inhibition has been already proposed in acute myeloid leukemia [24] and glioblastoma [12, 55], suggesting that p53-null cells are less sensitive to CK2 inhibitors. However, conflicting data have been reported on this point [56, 57]. We suppose that other hidden genetic traits might be shared by cells with similar sensitivity to CK2 targeting. Their identification will be important to plan the most appropriate clinical studies and precision medicine approaches based on CK2 targeting.

## Conclusions

To sum up, we strongly believe that the broad cancer essentiality of CK2 is far from having been disproved. Hopefully, newly discovered compounds, such as SGC-CK2-1 [10], the CX-4945 derivative 1c [50], and GO289 [58], will give new impetus to the investigations on this multipurpose anticancer target. In the meantime, premature conclusions about the dispensability of CK2 in cancer should not be considered consistent with the whole array of data available in the literature.

In this connection, it may be worthy to note that the purported dispensability of CK2 in non-cancer cells, grounded on the generation of viable C2C12 clones deprived of both CK2 catalytic subunits [59] has been subsequently challenged by the discovery in these cells of a CK2α’ C-terminally deleted mutant still able, despite its very low activity, to surrogate at least some of the CK2 functions [53]. This observation, in conjunction with the above mentioned, amply documented, abnormally high CK2 expression/activity found in a wide range of cancer cells, provide a strong, albeit indirect, argument (entirely independent of pharmacological implications) supporting the view that reliance on CK2 for survival is a general feature becoming especially stringent in tumor cells, possibly due to the well-known non-oncogene addiction phenomenon [5]. According to this scenario, a moderate decrease of endogenous CK2, still compatible with viability of non-cancer cells, may be lethal to tumor cells if it compromises functions that are essential for malignancy. Such a lethal/antiproliferative outcome, however, will also depend on a number of additional factors, besides the extent of CK2 decrement. One is the background of the tumors under scrutiny, which may rely on different mechanisms for their survival. Another, in the case of pharmacological treatments, is the nature of the CK2 inhibitor, which may affect to variable extents the phospho-proteome generated by CK2 [60]. This means that, coming back to the SGC-CK2-1 vs. CX-4945 issue, a comparative phospho-proteomics analysis with the two inhibitors is urgently needed in order to shed light on the controversial points dealt with in this perspective contribution.

## Data Availability

Data sharing is not applicable to this article, as no data sets were generated or analyzed during the current study.
